# Health-related quality of life and anxiety associated with childhood intermittent exotropia before and after surgical correction

**DOI:** 10.1186/s12886-021-02027-w

**Published:** 2021-06-30

**Authors:** Danyi Mao, Jing Lin, Lina Chen, Jiying Luo, Jianhua Yan

**Affiliations:** State Key Laboratory of Ophthalmology, Zhongshan Ophthalmic Center, Sun Yat-sen University, Guangzhou, 510080 Guangdong China

**Keywords:** Intermittent exotropia, Health-related quality of life, Hospital anxiety and depression scale

## Abstract

**Background:**

Intermittent exotropia (IXT) is the most common form of exotropia in children. In addition to cosmetic effects and loss of stereoscopic function, IXT may negatively impact the psychological well-being of children and their parents. The purpose of this study was to assess the patient-reported outcomes of Chinese children with IXT before and after strabismus surgery.

**Methods:**

The records of children with IXT who underwent strabismus surgery at the Zhongshan Ophthalmic Center of Sun Yat-sen University, China over the period from January 1, 2016 to December 31, 2018 were prospectively recruited. All children underwent ophthalmic and orthoptic examinations, including the prism and alternate cover test, fusion function by synoptophore, stereoacuity and Newcastle control score. Two patient-reported outcome measures were used: the intermittent Exotropia Questionnaire (IXTQ) to measure disease-specific health-related quality of life (HRQOL) and the Hospital Anxiety and Depression Scale (HADS) to measure anxiety and depression. Patient-reported outcome measurements were made before and after surgery with responses from children and their parents.

**Results:**

A total of 389 children were eligible for inclusion (47.8% male, 52.2% female, mean + SD age = 8.17 ± 2.81). Preoperative IXTQ scores in both children (48.21 ± 26.2) and their parents (44.6 ± 25.68) were significantly correlated with near stereoacuity (*P* = 0.029 and *P* = 0.015, respectively). The angle of deviation at near vision showed a negative linear relationship with visual function (*P* = 0.026) and psychological (*P* = 0.019) scores as well as opinions regarding surgery (*P* = 0.024). HADS scores (anxiety scale score: 11 ± 2.92, depression scale score: 10.44 ± 2.9) were also related to near stereoacuity (*P* < 0.05). After surgery, both children’s (74.83 ± 16.59) and parents’ (68.57 ± 17.06) IXTQ scores significantly improved (p<0.01). Children’s IXTQ scores were related to the angle of deviation at distance, and their psychological and visual function scores showed a negative relationship with the angle of deviation at near vision (*P* < 0.05).

**Conclusion:**

Children and parents’ HRQOL and HADS were associated with near stereoacuity. Parents usually attend more readily to the angle of deviation at near in their IXT children. HRQOL improved significantly after surgery and can be used as one of the indices for preoperative evaluation but is not recommended as a criterion for surgical intervention.

**Supplementary Information:**

The online version contains supplementary material available at 10.1186/s12886-021-02027-w.

## Background

Intermittent exotropia (IXT) is the most common form of exotropia in children [1]. The incidence in China ranges from 3.3 to 3.9% in the general population, which is much higher than that observed in Western populations [2–4]. In addition to cosmetic affects and loss of stereoscopic function [5], IXT may negatively impact the psychological well-being of children and their parents [6]. Health-related quality of life (HRQOL) represents an important tool for evaluating IXT patients, but it is rarely used by clinicians. When treating children with IXT, clinicians should focus not only on physiological parameters, such as the angle of deviation, Newcastle control score (NCS) and stereoacuity, but also on the psychological components assessed with HRQOL in these children and their parents. Surveys are currently available to perform these evaluations. For example, the Intermittent Exotropia Questionnaire (IXTQ) is a well-accepted, specific tool to assess HRQOL in IXT children and their parents [7]. In addition, the Hospital Anxiety and Depression Scale (HADS) provides a reliable tool for determining depression and anxiety status for hospital outpatient services and an effective means to measure the severity of emotional disorders [8]. With the use of these patient proxy scales, it is possible to achieve a more detailed assessment of the impact of IXT on the mental health of these children.

At present, only rarely are the psychological problems of children with IXT considered. In general, the more severe the IXT, the worse the HRQOL. However, Hatt and Lim [9, 10] found that whereas parents’ HRQOL was related to children’s IXT severity, no such relationship was present in the children. In this way, the clinical findings of IXT in these children do not fully correspond with their mental health. Another indication of this difference between the children and their parents was the finding that parents with poorer HRQOL scores were more likely to make the decision to perform surgery [11]. Chiu et al. [12] suggested that HRQOL should be incorporated into postoperative evaluations. In support of this proposal are the results of McKenzie et al., who reported that IXT children, especially boys, are three times more likely to suffer from mental illness in the future [13].

As no clear criteria currently exist for surgical intervention in childhood IXT, this raises the issue as to whether HRQOL should be included as an indication for surgery as well as one of the criteria for evaluating surgical success. Although there are a few papers that have reported on anxiety and depression in adults with strabismus [14–16], to the best of our knowledge, no information regarding these factors is available in children with IXT and their parents. Therefore, the purpose of this study was to assess both the HRQOL and the HADS in a large sample of IXT children and their parents related to the severity of the IXT. In addition, we reexamined HRQOL after surgery to examine the relationship between cosmetic/functional recovery and HRQOL.

## Methods

### Patients

Children (*N* = 389) with IXT who underwent strabismus surgery at the Zhongshan Ophthalmic Center of Sun Yat-sen University, China over the period from January 1, 2016 to December 31, 2018 were recruited for this study. The following inclusion criteria were employed: (1) 5–17 years old, (2) basic type (the deviation was within 10Δ at distance and near), (3) angle of distant exodeviation ≥15 prism diopters (PD), (4) no “A” or” V” pattern or vertical deviation, (5) best corrected visual acuity of no less than 20/30 and bilateral difference of not greater than two lines (for statistical analysis, the visual acuity would convert to logMAR.) and (6) no abnormality in the anterior segment and fundus. The exclusion criteria included (1) a history of ophthalmic and/or strabismus surgery or botulinum injection, (2) previous vision training, (3) nystagmus, paralytic or restrictive exotropia, developmental delay and/or any learning disability and (4) any other neurological and/or psychological disorders. Informed consent was obtained from all patients and their parents. The study was compliant with the Declaration of Helsinki, and additional approval was obtained from the Research Ethics Board of the Zhongshan Ophthalmic Center of Sun Yat-sen University, China (approval NO. 2020KYPJ068).

### Clinical evaluation and surgical treatment

All participants had undergone a comprehensive eye examination, including best corrected visual acuity, intraocular pressure, cycloplegic refraction, anterior segment examination by slit-lamp and fundus colour photography. The visual acuity was converted to logMAR. Orthoptic examinations included the prism and alternate cover test (PACT) at distance (6 m) and near fixation (33 cm), fusion function by synoptophore, near stereoacuity (33 cm) by Titmus test, distance stereoacuity (5 m) by Randot test, NCS and the Worth 4 dot test. All patients underwent strabismus surgery under general anaesthesia. The surgical indication for IXT is that there is either definite evidence of existing defective binocular vision or gradual loss of fusional control. For the basic type of IXT, either bilateral rectus recession of the lateral rectus or unilateral recession of the lateral rectus and resection of the medial rectus of the nondominant eye is the right choice of surgical procedure. However, we prefer unilateral surgery. For cases with less than 25 PD of exotropia, we usually perform only unilateral recession of the lateral rectus. A total of 69.9% of patients underwent unilateral lateral rectus recession and medial rectus resection, and 10% underwent bilateral lateral rectus recession, whereas 20.1% underwent unilateral lateral rectus recession. After a minimum period of 6 months of follow-up, exotropia of ≤10 PD and esotropia ≤5 PD were considered surgical success. Normal sensory fusion was classified as a patient who had simultaneous perception, fusion faculty and stereoacuity, and if any one of those functions was impaired, it was classified as abnormal.

### HADS

The 14 items comprising the HADS serve to measure anxiety and depression symptoms, with seven items for the anxiety scale (HADS anxiety) and seven for the depression scale (HADS depression) [17] (Additional file 1). The scoring method consists of 0-never, 1-rarely, 2-sometimes, 3-often and 4-almost always. Items from each of the 7-item anxiety and depression subscales were summed to generate a total subscale score. Total subscale scores of 7 or less indicate non-cases, 8–10 indicate borderline and scores of ≥11 indicate definite cases [18]. It has been reported that the Chinese version of the HADS possesses excellent reliability and validity [19]. Parents were asked to read each statement and choose the answer that best described how they had felt in the preceding week [20]. The HADS was performed one day before the surgery.

### IXTQ

The IXTQ has three components: a self-report of the child’s own HRQOL completed by the child (5–17 years old), the Proxy IXTQ and a report of the child’s HRQOL as completed by the parent [21]. An additional scale for the children’s psychological and visual function and surgical opinions was completed by the parents. Each item had five response options: 100-Never, 75- Almost never, 50-Sometimes, 25-Often and 0-Almost always. For children aged 5–7 years, only three opinions were included: 100-not at all, 50-sometimes and 0-a lot. The IXTQ score for each child and their parents was calculated and ranged from 0 (worst) to 100 (best) HRQOL. During the HRQOL assessment, the children and their parents answered questionnaires separately without any verbal or nonverbal communication. Questionnaires were self-administered with written instructions and were supervised by the same investigator (QL). If the child or his/her parents had problems understanding the question, a verbal interview was conducted without any explanation or elaboration [6, 7, 22]. The IXTQ is available online in an administrable format with user instructions: https://public.jaeb.org/pedig/view/Reference. It was performed one day before the surgery and again at the final follow-up visit.

All the questionnaires used in this study were translated by the author using standardized procedures. The Chinese intermittent exotropia questionnaire was translated by two bilingual experts who were fluent in both Chinese and English. With only minor modifications, the Chinese questionnaire was consistent with the original questionnaire. Two English teachers from the United Kingdom then compared the reverse translation questionnaire with the original English version and found no significant differences.

### Statistical analysis

Independent samples t-tests were used to compare the pre- and postoperative differences in HRQOL results between children of different ages and genders and their parents. Children with differences in stereo function and stereoscopic function were compared using one-way analysis of variance. Relationships among children, proxy HRQOL scores, HADS scores and severity of IXT were evaluated using multivariate linear regression analyses and Kendall’s tau-b analysis. The SPSS 19.0 software package (SPSS Inc., Chicago, IL, USA) was used to perform these statistical analyses. A *P*-value of ≤0.05 was required for results to be considered statistically significant.

## Results

### Demographic data

Of the 389 children included in this study, 47.8% were male and 52.2% were female. Their ages ranged from 5 to 17 years old (Mean + SD = 8.17 ± 2.81). The best corrected visual acuity was − 0.05 ± 0.21 (− 0.1–0.2) in the right eye and − 0.05 ± 0.1 (0–0.2) in the left eye. The spherical equivalent of the right eye ranged from − 8.5 D to + 9.25 D (− 0.08 ± 2.38 D) and from − 10.5 D to + 8.25 D (− 0.03 ± 2.32 D) in the left eye (Table 1).

**Table 1:** Clinical characteristics of childhood intermittent exotropia

Clinical characteristics			N = (389)
Sex (Male/Female)			186/203
Age (years, mean ± SD)			8.17 ± 2.81(5–17)
VA of eyes (mean ± SD)	Left		−0.05 ± 0.21(0.1–0.2)
	Right		−0.05 ± 0.1(0–0.2)
Deviation (PD, mean ± SD)	Near		31.08 ± 9.92(10–64)
	Distance		31.43 ± 8.94(12–64)
Sensory fusion	Normal (III)		82(21%)
	Abnormal (Nil-II)		307(79%)
Stereoacuity (seconds)	Near	Good (≤63″)	116(29.8%)
		Moderate(≤200″)	79(20.3%)
		Poor(> 200″)	194(49.9%)
	Distance	Good (≤63″)	25(6.4%)
		Moderate(≤200″)	41(10.5%)
		Poor(> 200″)	323(83.1%)
Near control score (median,range)			9(7–9)

*PD* prism diopter; *VA* visual acuity

### Severity of IXT and IXTQ scores

**Fig. 1:**
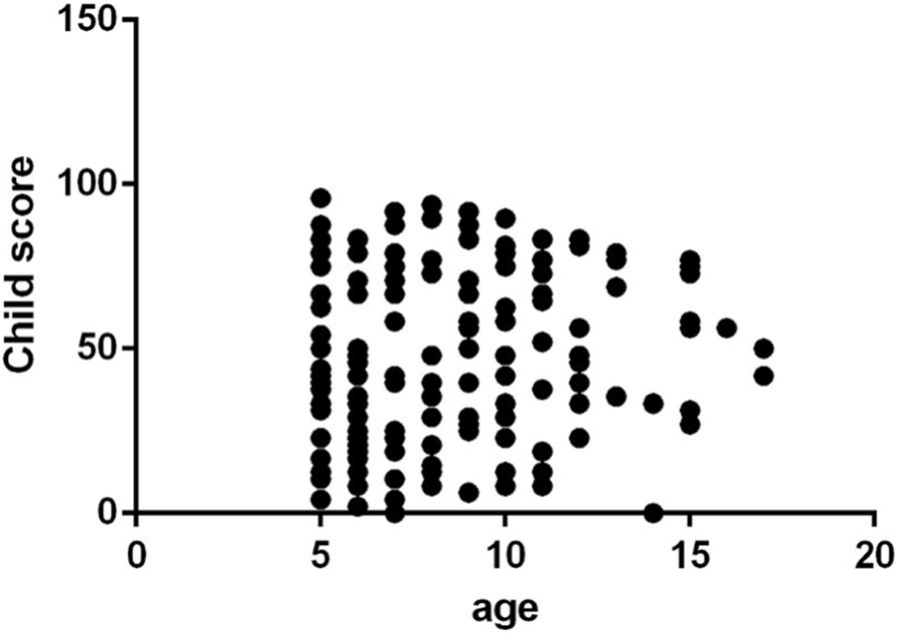
Age was not correlative with the preoperative child HRQOL(*P* = 0.68)

**Fig. 2:**
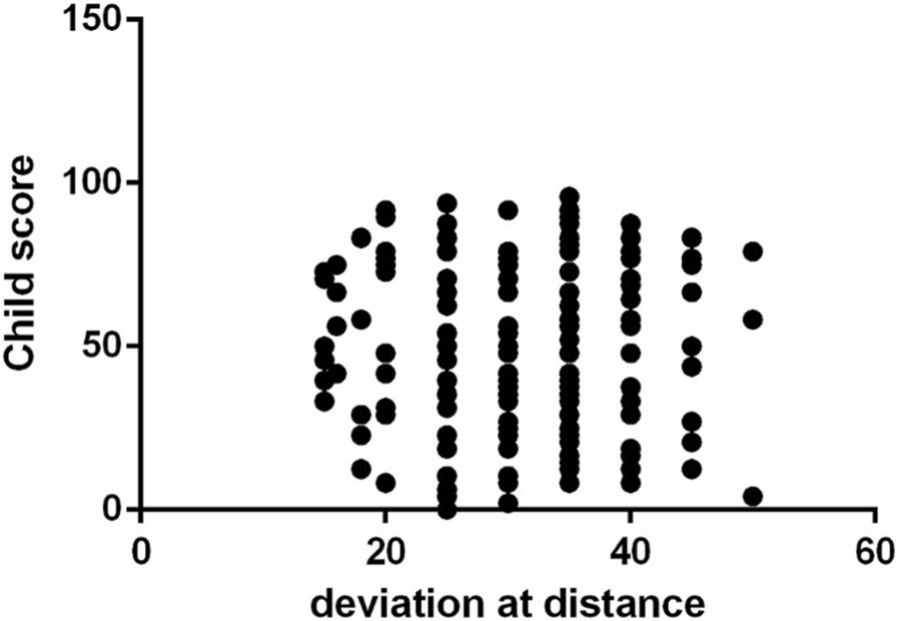
The angle of deviation at distance was not correlative with the preoperative child HRQOL(P = 0.37)

**Fig. 3:**
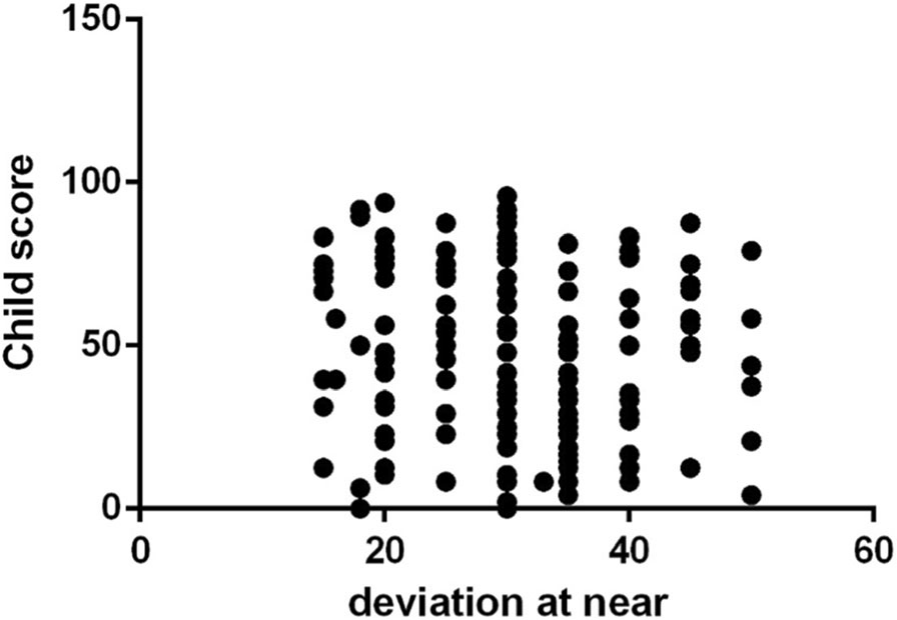
The angle of deviation at near was not correlative with the preoperative child HRQOL(P = 0.497)

The mean + SD IXTQ score in children with IXT was 48.21 ± 26.2, and their proxy score was 44.6 ± 25.68. The average score for psychological function, visual function and surgical opinions was 42.04 ± 20.16. The children’s IXTQ scores were related to the proxy and comprehensive scales (*P* < 0.01) as well as to near stereoacuity (β = − 6.41, *P* = 0.029). There were no significant differences in HRQOL between children of different ages, angles of deviation or fusion functions (Figs. 1, 2 and 3). In the parent’s scale, poor near stereoacuity was associated with lower scores (β = − 6.889, *P* = 0.015). The angle of deviation at near showed negative linear relationships with visual function (β = − 1.05, *P* = 0.026) and psychological scores (β = − 1.124, *P* = 0.019) as well as opinion regarding surgery (β = − 1.146, *P* = 0.024) (Table 2).

**Table 2:** Multivariate linear regression of the IXTQ and severity of IXT

Examination of IXT		Child score	Proxy score	Visual Function subscale	Psychological subscale	Surgery subscale
		48.21 ± 26.2	44.6 ± 25.68	43.15 ± 21.98	41.7 ± 22.32	42.22 ± 23.71
Deviation	Near	*P* = 0.497	*P* = 0.422	P = 0.026*	P = 0.019*	P = 0.024*
		β = −3.86	β = −0.362	**β = −1.05**	**β = − 1.124**	**β = − 1.146**
	Distance	*P* = 0.370	*P* = 0.114	*P* = 0.967	*P* = 0.773	*P* = 0.657
		β = 0.53	β = 0.908	β = 0.016	β = 0.112	β = 0.183
Sensory fusion		*P* = 0.837	*P* = 0.529	*P* = 0.388	*P* = 0.133	*P* = 0.960
		β = 1.558	β = 1.050	β = −1.223	β = 2.165	β = 0.076
Stereoacuity	Near	P = 0.029*	P = 0.015*	*P* = 0.521	*P* = 0.235	*P* = 0.274
		**β = −6.41**	**β = − 6.889**	β = −1.530	β = −2.879	β = 2.811
	Distance	*P* = 0.221	*P* = 0.412	*P* = 0.983	*P* = 0.635	*P* = 0.655
		β = 3.033	β = 0.293	β = −0.042	β = 0.980	β = −9.979
Near control score		*P* = 0.965	*P* = 0.061	*P* = 0.448	*P* = 0.372	*P* = 0.940
		β = 0.021	β = −0.598	β = − 0.205	β = − 0.245	β = − 0.022

Both the child’s and proxy scores were related to near stereoacuity. Subscales of visual function, psychological and surgical options were related to the angle of deviation at near. IXTQ: Intermittent Exotropia Questionnaire

### Severity of IXT and anxiety and depression scores

The mean + SD anxiety and depression score of the parents was 11.2 ± 2.92, of which an anxiety scale score of ≥8 accounted for 95.87%, whereas a depression scale score of ≥8 accounted for 92.01%. The anxiety (R = -0.215, *P* = 0.002) and depression (R = -0.182 *P* = 0.009) scores showed a negative linear relationship with near stereoacuity. There were no statistically significant differences in other results of the IXT examination among the groups (Table 3).

**Table 3:** Relationship between the severity of IXT and HADS

Kendall’s tau-b analysis		Anxiety scale score	Depression scale score
		11.2 ± 2.92	10.44 ± 2.9
Deviation	Near	*P* = 0.483	*P* = 0.072
	Distance	*P* = 0.163	*P* = 0.946
Sensory fusion		*P* = 0.780	*P* = 0.756
Stereoacuity	Near	**P = 0.002**	**P = 0.019**
	Distance	*P* = 0.851	*P* = 0.359
Near control score		*P* = 0.293	*P* = 0.811

HADS scores were negatively correlated with near stereoacuity. HADS: Hospital Anxiety and Depression Scale

### Surgical outcomes and postoperative HRQOL

Postoperative orthoptic measurements showed that 275 patients (70.6%) achieved successful surgical results. Fifty-one patients (13.3%) were overcorrected, and 63 patients (16.1%) were undercorrected (Table 4).

**Table 4:** Clinical characteristics after surgery

Clinical characteristics			N = (389)
Deviation at distance (PD, mean ± SD)	Overcorrected		11.49 ± 6.17
	Successful		−1.69 ± 3.75
	Undercorrected		−14.6 ± 3.29
Sensory fusion	Normal (III)		120(30.8%%)
	Abnormal (Nil-II)		296(69.2%)
Stereoacuity (seconds)	Near	Good (≤63″)	121(31.1%)
		Moderate(≤200″)	108(27.7%)
Sensory fusion		Poor(> 200″)	160(41.1%)
	Distance	Good (≤63″)	21(5.3%)
		Moderate(≤200″)	36(9.25)
		Poor(> 200″)	332(85.3%)

*PD* prism diopter; *VA* visual acuity

Overcorrected:esotropia > 5PD

Successful: exotropia of ≤10PD and esotropia ≤5PD

Undercorrected: exotropia>10PD

All HRQOL scores in IXT children were higher after surgery than their preoperative scores (*P* < 0.05). The mean + SD IXTQ score in these children was 74.83 ± 16.59, and the proxy IXTQ score was 68.57 ± 17.06. The overall mean + SD score of the children’s psychological, visual function and surgical opinions was 50.28 ± 20.29 (Table 5). Even in patients with over- and undercorrections (exotropia > 10 PD, esotropia > 5 PD), postoperative IXTQ scores were significantly improved (*p* = 0.015). After surgery, the children’s IXTQ score showed a negative linear relationship with the angle of deviation at distance (β = − 0.599, *P* = 0.039), whereas no significant correlations in parental IXTQ scores were obtained with any of the clinical features. Visual function (β = − 0.856, *P* = 0.036) and psychological scores (β = − 1.0, *P* = 0.012) displayed a negative linear relationship with the angle of deviation at near vision (Table 6).

**Table 5:** Pre- and post-operative IXTQ scores in childhood intermittent exotropia

IXTQ	Preoperative	Postoperative	***P*** values
Child score	48.21 ± 26.2	74.83 ± 16.59	**0.001**
Proxy score	44.6 ± 25.68	68.57 ± 17.06	**0.001**
Average score	42.04 ± 20.16	50.28 ± 20.29	**0.001**

Differences between pre- and post-operative IXTQ scores were statistically significant

**Table 6:** Multivariate linear regression of surgical outcomes and post-operative IXTQ

Examination of IXT		Child score	Proxy score	Function subscale	Psychological subscale	Surgery subscale
		74.83 ± 16.59	68.57 ± 17.06	60.03 ± 44.60	53.65 ± 24.32	37.16 ± 21.43
Deviation	Near	*P* = 0.912	*P* = 0.215	P = 0.036*	P = 0.012*	*P* = 0.360
		β = 0.036	β = −0.415	**β = −0.865**	**β = −1.000**	β = −0.353
	Distance	P = 0.039*	*P* = 0.707	*P* = 0.406	*P* = 0.135	*P* = 0.051
		**β = −0.599**	β = − 0.129	β = − 0.302	β = 0.608	β = − 0.770
Sensory fusion		*P* = 0.473	*P* = 0.789	*P* = 0.701	P = 0.529	*P* = 0.057
		β = −0.580	β = − 0.221	β = 0.391	β = − 0.618	β = 2.104
Stereoacuity	Near	*P* = 0.542	*P* = 0.606	*P* = 0.265	*P* = 0.746	*P* = 0.853
		β = −0.800	β = − 0.691	β = 1.841	β = 0.516	β = 0.331
	Distance	*P* = 0.878	*P* = 0.594	*P* = 0.508	*P* = 0.065	*P* = 0.731
		β = −0.278	β = 0.986	β = −1.509	β = −4.062	β = 0.205
Near control score		*P* = 0.835	*P* = 0.119	*P* = 0.144	*P* = 0.632	*P* = 0.621
		β = −0.091	β = 0.700	β = 0.807	β = −0.165	β = −0.173

Children’ IXTQ scores were related to the angle of deviation at distance. Psychological and visual function scores were negatively related to the angle of deviation at near. No statistically significant correlations were obtained between parental IXTQ scores and any of the clinical features

## Discussion

Attempts at establishing a relationship between the child’s HRQOL and clinical severity of their IXT have proven inconsistent based on previous literature. A multiethnic paediatric eye disease study (MEPEDS) [23] surveyed children with strabismus at 25 to 72 months of age and found that strabismus reduced the HRQOL in these preschool children. However, in that study, a wide variety of strabismus types were included in the analysis. In Wang et al.’s study, the deviation angle at distance and exotropia control at home were associated with the child’s HRQOL [22], whereas Lim et al. believed that the child’s HRQOL and clinical severity were not related [10]. Our current results were mixed; that is, the child’s IXTQ was related to near stereoacuity but not to the deviation angle, NSC or distant stereoacuity. The clinical findings partially correspond with the child’s HRQOL responses. Children’s perception of strabismus differs from that of adults [24], in part due to the limited attention that children direct to their visual problems [25]. In particular, younger children have not yet formed a clear aesthetic concept and an established awareness of their eye disease. Essentially, strabismus is their norm and has little effect on their daily life [26]. Moreover, exotropia in children with IXT appears intermittently, which may also make it difficult for them to consistently respond to this condition and would thus not always affect their quality of life. In contrast, stereoscopic function is more critical for detailed near works, and if disturbed, it may lead to children’s inability to perform delicate operations [27, 28] and affect their social activities [29]. With poor stereopsis, it is much harder to judge where objects are in space and estimate the distance between one’s hand and the object. An easy task, such as catching a ball or taking steps, may become difficult for these children [30, 31]. With no stereopsis, people walk 10% slower than normal [32]. Patients with IXT experience an initial decline in distance stereoacuity followed by a decline in near stereoacuity. Before the impairment of near stereoacuity, those problems have not yet shown up, and as IXT worsens, the problems become increasingly serious. Our findings that children in the later stages of IXT show effects on their HRQOL may be related to the disturbance of stereoscopic function.

Parents’ proxy scale scores were also related to near stereoacuity, and the subscale scores of psychological, visual function and surgery opinion had a negative relationship with the deviation angle at near. These results indicate that not only do the later stages of IXT affect the parents’ HRQOL but also that deviations at near represent their greatest concern. As near deviation can be readily detected by parents, it seems understandable that they would worry that this IXT affects their children’s physical and mental health. These parents would observe the frequency and severity of their children’s near deviation and, therefore, be more inclined to take their children to see a doctor as well as be more concerned about the surgical risk and surgical prognosis. Wang et al. [22] found that both the child’s and their parents’ HRQOL showed a trend towards> correlating with clinical severity, with large deviation, poor control and poor stereo function being significantly associated with higher IXTQ scores. In our experience, we found that some patients with a more serious IXT may show a lower HRQOL score, which then predisposes them to ignore the severity of IXT and thereby delays the time for surgery. In fact, the relationship between the angle of deviation, stereoacuity and fusion function is not yet clearly defined [33], but the larger the angle of deviation is, the more likely it will be to disrupt the binocular balance [34]. A loss of stereoacuity in children may result in abnormal daily activity that is readily apparent. Overall, the objective clinical findings of IXT do not provide a good representation for the subjective understanding of the disease in these children. Preoperative HRQOL is related to the later stages of impaired stereoacuity in children with IXT and may provide a slight guide for clinicians to judge the severity of IXT and the time for surgical intervention. However, these HRQOL scores are not in full accord with the severity of IXT. Therefore, it is important for clinicians to inform parents about all aspects of IXT, including the various clinical features and surgical versus nonsurgical interventions, that may help them to make the appropriate decision.

The results of the anxiety and depression scores from parents indicate that they exhibit an obvious tendency for anxiety and depression regarding their children’s IXT. It is clear that the parents are truly concerned about their children with IXT, as more than 90% of them show a decline in mental health. We found that the decline in near stereoacuity can result in a significant amount of anxiety and depression, although there is no correlation with other factors, such as deviation angle, NSC and distance stereoacuity. It has been reported that visual impairment and loss of binocular function are related to symptoms of anxiety and depression [35]. Decreasing near stereoacuity results in deterioration in reading abilities and academic performance [36, 37], and reduced stereoacuity function also limits career options for the future [31]. Some of them may avoid performing activities that may bring attention to their defects [38]. With the decline in binocular function in the later stages of IXT, these children can face poor academic performance and a negative attitude towards strabismus by their peers. These problems may cause anxiety or depression in their parents. The results from a prospective study by Chai et al. [39] revealed that children, adolescents or adults with strabismus may experience symptoms of anxiety and depression and that children are more affected and less likely to recover their emotional and mental state. Mcbain [16] and Snaith [40] emphasized that it was society’s and patient’s awareness of the disease that affected the quality of life, not the severity of the disease itself. Our results also confirm this finding. Almost all parents show signs of psychological stress in response to the various degrees of IXT severity in their children, which can explain their anxiety and likelihood of taking their children to the hospital for treatment.

The postoperative HRQOL in both children and their parents significantly improved, likely due to the changes in the child’s appearance after surgery [41]. In fact, all subscales of IXTQ scores show improvements after surgery. It is generally believed that both the cosmetic and the functional recovery of binocular vision following surgery result in a positive impact on the social skills, emotions and mental state of these children. These postoperative HRQOL scores showed a significant relationship with the eye position, and even in patients with under- or overcorrections resulting from strabismus surgery, their postoperative HRQOL scores were substantially improved. Interestingly, these changes in psychological state may not parallel the surgical outcome. For example, Mruthyunjaya et al. [42] noted that subjective satisfaction can be obtained when the eye position is within 10 PD after surgery, even if it is objectively considered unsuccessful by the surgeon. Archer et al. [43] also reported that although strabismus surgery in children can improve their HRQOL, there was no statistically significant difference between successful and unsuccessful surgical outcomes in patients. Another explanation was that as a parent, they would evaluate the quality of life of their own child as significantly more highly after low-risk strabismus surgery, regardless of any functional outcome. This is similar to the findings of our study. Strabismus surgery can produce a similar degree of comfort and reduce the patient’s concern about their disease as that in response to a placebo. After surgery, children’s HRQOL scores were consistent with those of their parents. Perhaps the surgery enables both the children and their parents to attend similarly to the IXT.

There are certain limitations in this study. Currently, no unified criteria exist for the judgement of IXT severity. Different methods used for measuring stereoacuity and fusion function may reveal quite different results. In addition, there were a few children in this study with a history of amblyopia treatment, and some wore glasses, which may affect their HRQOL. Finally, we did not have a healthy control group, and the fact that all patients in our study underwent strabismus surgery may introduce some bias with regard to the whole population of children with IXT.

## Conclusion

Children’s and parents’ HRQOOL scores and anxiety and depression scores were associated with near stereoacuity. The improvements in HRQOL scores in both the children and the parents following surgery indicate that timely surgery is important for enhancing the quality of life of these children. However, HRQOL scores failed to reflect the seriousness of childhood IXT, nor could these scores be used as an indication for surgical intervention. It is important to note that HRQOL scores vary widely between people as well as with the same illness within an individual over time, as these scores are based on personal assessments and are influenced by physical, psychological and social factors [44]. Nonetheless, these HRQOL scores should be considered by the clinician when evaluating children with IXT to understand the anxiety of these children and their parents and attend to their psychological state.

## Supplementary Information

**Additional file 1.** Hospital Anxiety and Depression Scale (HADS). The 14 items comprising the HADS serve to measure anxiety and depression symptoms, with seven items for the anxiety scale (HADS anxiety) and seven for the depression scale (HADS depression).

## Data Availability

The datasets used and/or analysed during the current study are available from the corresponding author on reasonable request.

## Abbreviations

HRQOL: Health-related quality of life
HADS: Hospital anxiety and depression scale
IXT: Intermittent exotropia
IXTQ: Intermittent Exotropia Questionnaire
